# Development and Validation of a New Lymph Node Ratio-Based Staging System for Ampullary Carcinoma After Curative Pancreaticoduodenectomy

**DOI:** 10.3389/fonc.2021.811595

**Published:** 2022-01-20

**Authors:** Xiaojie Zhang, Chongyuan Sun, Zefeng Li, Tongbo Wang, Lulu Zhao, Penghui Niu, Chunguang Guo, Yingtai Chen, Xu Che, Dongbing Zhao

**Affiliations:** ^1^ Department of Pancreatic and Gastric Surgical Oncology, National Cancer Center/National Clinical Research for Cancer/Cancer Hospital, Chinese Academy of Medical Sciences and Peking Union Medical College, Beijing, China; ^2^ Department of Hepatobiliary and Pancreatic Surgery, National Cancer Center/National Clinical Research Center for Cancer/Cancer Hospital & Shenzhen Hospital, Chinese Academy of Medical Sciences and Peking Union Medical College, Shenzhen, China

**Keywords:** ampullary adenocarcinoma, lymph node metastasis, lymph node ratio (LNR), SEER, prognosis, staging system

## Abstract

**Background:**

Lymph node metastasis (LNM) is closely associated with the prognosis of ampullary carcinoma (AC). The purpose of this study is to explore the relationship between lymph node ratio (LNR) and the prognosis of patients with AC after curative pancreaticoduodenectomy and to establish a new LNR-based staging system.

**Methods:**

AC patients in the Cancer Hospital, Chinese Academy of Medical Sciences, between 1998 and 2020 were retrospectively reviewed as the training cohort; and AC patients in the Surveillance, Epidemiology, and End Results (SEER) database between 2010 and 2018 were obtained as the validation cohort. Within the training group, Kaplan–Meier survival analyses and Cox proportional hazards regression were conducted to assess the prognostic value of LNR and establish a new LNR-based staging system. Then, the new staging system was compared with the 8th American Joint Committee on Cancer (AJCC) TNM staging system in both the training and validation cohorts.

**Results:**

A total of 264 patients in the training cohort and 199 patients in the validation cohort were enrolled. Significant overall survival (OS) difference was observed between LNR-low stage and LNR-high stage in both training (*p* = 0.001) and validation cohorts (*p* < 0.001). Then a new LNR-based staging system was developed. Under the new system, the number of patients in the training cohort and validation cohort of stage I, stage II, and stage III was 30 (11%) vs. 18 (9%), 190 (72%) vs. 96 (48%), and 44 (17%) vs. 85 (43%), respectively. The new staging system classified patients with respect to survival better than did the 8th AJCC TNM staging system.

**Conclusions:**

The new LNR-based staging system had better discriminability for predicting survival in AC patients after curative pancreaticoduodenectomy. More data are needed for further validation.

## Introduction

Ampullary carcinoma (AC) is a relatively rare tumor arising from the ampulla of Vater, with an incidence of around 0.6 cases in 100,000 people (1–3). Generally, AC patients are diagnosed at an early stage due to early symptoms of biliary obstruction (4, 5). At present, curative pancreaticoduodenectomy (Whipple) remains the mainstay of treatment for AC. It has been reported the rate of lymph node metastasis (LNM) ranges from 20% to 50% (1). Despite many clinicopathologic characters are associated with prognosis for AC patients, LNM is still one of the most crucial risk factors (1, 6–25).

The number of involved lymph nodes occupies a significant part in the current TNM Classification of Malignant Tumors (TNM) staging system proposed by the American Joint Committee on Cancer (AJCC). Nevertheless, debates still exist about the node stage for AC patients. Previous studies showed some differences when conducting subgroup analysis according to the number of LNM (1, 9, 13, 15, 19, 21). Recently, a retrospective study enrolled 111 patients who underwent Whipple surgery found that patients with ≥3 local LNMs had similar survival time as compared with the patients with distant LNM (1). Moreover, the total number of harvested lymph nodes is also a vital prognostic factor in AC (8, 12, 15, 17). In view of this, some studies evaluated node staging for AC based on lymph node ratio (LNR) (7, 9, 11, 18, 23). Yet the cutoff value of LNR varied across studies. Moreover, whether the LNR could replace the current N staging has not been evaluated.

Therefore, in the present study, we assessed the prognostic value of the total harvested number of lymph nodes and LNR for AC patients after curative Whipple surgery. Additionally, we conducted a comparative analysis of the current lymph node categories of AC in the eighth edition of the AJCC staging guidelines. Furthermore, we used a separate cohort from the Surveillance, Epidemiology, and End Results (SEER) database for further external validation.

## Materials and Methods

### Patients

Clinicopathologic data of ampullary adenocarcinoma patients who underwent curative pancreaticoduodenectomy in the Cancer Hospital, Chinese Academy of Medical Sciences, between 1998 and 2020 were retrospectively collected as the training cohort. We then excluded some patients according to the following criteria: i) patients diagnosed with neuroendocrine tumors, adenoma, and other rare tumor types; ii) patients with zero regional lymph node examined; iii) death within a month after the operation due to complications and other reasons; and iv) patients with missing or incomplete clinicopathologic information. In total, 264 patients were enrolled in the training cohort.

Persistent data of AC patients in the SEER database between 2010 and 2018 were obtained as validation cohort using the SEER * State v8.3.6 tool on July 8, 2021. Selection items were as follows: i) primary site—labeled = “C24.1-Ampulla of Vater”; ii) ICD-O-3 Hist/behav = “/3: adenocarcinoma, NOS”; and iii) diagnostic confirmation = “Microscopically confirmed.” The main exclusion criteria were as follows: i) AC was not the first primary malignant tumor; ii) patients did not receive pancreaticoduodenectomy; iii) patients with zero regional lymph node examined; and iv) some important information was unknown, such as staging, tumor size, number of regional lymph nodes examined, and number of LNM. Eventually, a total of 199 patients were included in the validation cohort.

### Covariates and Outcomes

The major covariates include gender, age, preoperative jaundice, intraoperative transfusion, operation time, tumor size, differentiation, number of regional lymph nodes examined, LNR, AJCC TNM stage (8th edition), blood vessel invasion, postoperative complications, and adjuvant treatment. LNR was defined as the ratio of the number of LNM to the total number of regional lymph nodes examined.

Follow-up data in the training cohort were collected through telephone and outpatient reexaminations. In total, 62 patients were lost to follow-up, and the follow-up rate was 76.5%. The primary outcome was overall survival (OS), which was defined as the time interval from diagnosis to the most recent follow-up date or date of death. The second outcomes were disease-free survival (DFS) and cancer-specific survival (CSS). DFS was defined as the time from surgery to the local recurrence or distant metastasis. CSS was calculated as the time from diagnosis to the most recent follow-up date or date of death caused by AC.

### Statistical Analysis

To determine the optimal cutoff values, we used X-tile software (version 3.6.1) and converted the continuous variables into categorical variables before conducting statistical analyses. Univariable survival analysis was conducted using the training cohort according to the Cox proportional hazards regression model. Then covariates with *p* < 0.2 were included in Cox multivariate regression to find independent prognostic factors. Hazard ratios (HRs) and their 95% CI were presented.

Based on the survival analysis results, we developed a new LNR-based staging system for AC after curative Whipple surgery. The prognostic performances of the novel staging and the 8th AJCC TNM stage were compared in the training cohort and validation cohort with OS, DFS, and CSS as outcomes.

All statistical analyses were conducted with IBM SPSS statistics 21 (IBM Corp, Armonk, NY, United States). The GraphPad Prism software (version 8.0.2) was utilized to generate survival curves. A two-sided test with *p* ≤ 0.05 was considered to be statistically significant.

## Results

### Demographic and Clinicopathologic Data

A total of 264 patients in the training cohort and 199 patients in the validation cohort were enrolled. The detailed demographic and clinicopathologic data were depicted in **Table 1**. The median number of total regional examined lymph nodes in the training and validation cohorts was 11 (interquartile range (IQR): 7–18) and 15 (IQR: 11–20), respectively. LNM occurred in 29.5% AC patients in the training cohort, while in 66.3% AC patients in the validation cohort. Furthermore, the number of N1 stage and N2 stage in the training and validation cohorts was 25% vs. 44.7%, and 4.5% vs. 21.6%, respectively. The median of LNR in the training and validation cohorts was 0.00 (IQR: 0.00–0.05) and 0.08 (IQR: 0.00–0.21), respectively. According to the X-tile analysis results, LNR was divided into three groups: LNR = 0, 0 < LNR ≤ 0.1, and LNR > 0.1 (**Supplementary Figure 1**). In the LNR = 0 group, all the patients were at the N0 stage. In the 0 < LNR ≤ 0.1 group, all the patients were at the N1 stage. In the LNR > 0.1 group, 32/44 patients were at the N1 stage and 12/44 patients at the N2 stage in the training cohort, while 44/87 patients were at the N1 stage and 43/87 patients at the N2 stage in the validation cohort.

**Table 1:** The clinicopathologic characteristics of the AC patients in the training and validation cohorts.

Characteristic	Training cohort		Validation cohort	
	N = 264	%	N = 199	%
Sex				
Male	147	55.7%	114	57.3%
Female	117	44.3%	85	42.7%
Age				
≤47	58	22.0%	14	7.0%
48–68	173	65.5%	103	51.8%
≥69	33	12.5%	82	41.2%
Preoperative biliary drainage				
No	207	78.4%		
Yes	57	21.6%		
Intraoperative transfusion				
No	122	46.2%		
Yes	142	53.8%		
Operation time				
≤6 h	190	72.0%		
>6 h	74	28.0%		
Tumor size				
≦2.7 cm	177	67.0%	139	69.8%
>2.7 cm	87	33.0%	60	30.2%
Differentiation				
Poor	96	36.4%	71	35.7%
Moderate	113	42.8%	107	53.8%
Well	55	20.8%	21	10.6%
Regional nodes examined [median, IQR]	11 [7–18]		15 [11–20]	
≤6	58	22.0%	15	7.5%
>6	206	78.0%	184	92.5%
LNR [median, IQR]	0.00 [0.00–0.05]		0.08 [0.00–0.21]	
0	186	70.5%	67	33.7%
≤0.1	34	12.9%	45	22.6%
>0.1	44	16.7%	87	43.7%
T stage				
T1	30	11.4%	18	9.0%
T2	103	39.0%	54	27.1%
T3	131	49.6%	127	63.8%
N stage				
N0	186	70.5%	67	33.7%
N1	66	25.0%	89	44.7%
N2	12	4.5%	43	21.6%
TNM stage				
I	113	42.8%	33	16.6%
II	74	28.0%	34	17.1%
III	77	29.2%	132	66.3%
Blood vessel invasion				
No	201	76.1%		
Yes	63	23.9%		
Postoperative complications				
No	161	61.0%		
Yes	103	39.0%		
Adjuvant treatment				
No	176	66.7%		
Yes	61	23.1%		
Unknown	27	10.2%		

AC, ampullary carcinoma; IQR, interquartile range; LNR, lymph node ratio.

### Survival Outcomes

**Figure 1:**
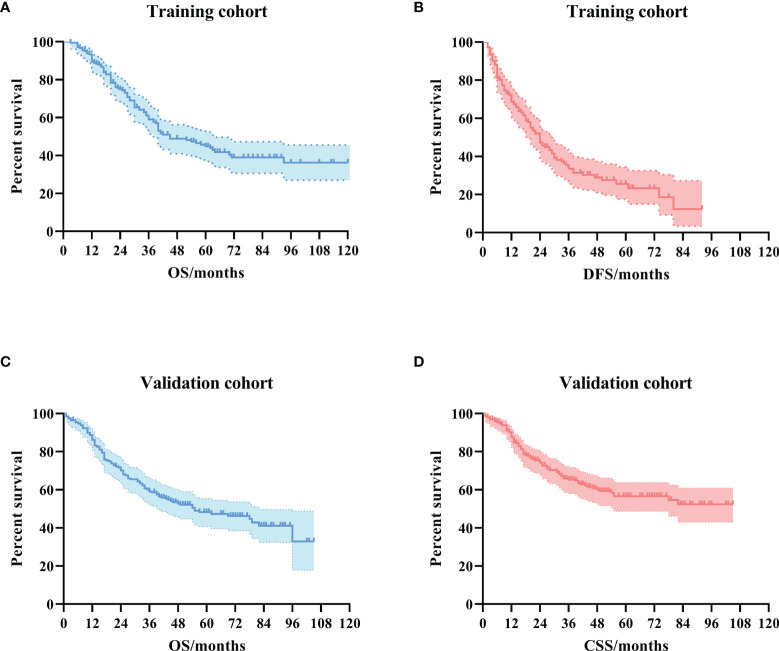
The Kaplan–Meier survival curves for ampullary carcinoma patients after curative pancreaticoduodenectomy. **(A)** OS curve in the training cohort. **(B)** DFS curve in the training cohort. **(C)** OS curve in the validation cohort. **(D)** CSS curve in the validation cohort. OS, overall survival; DFS, disease-free survival; CSS, cancer-specific survival.

In the training cohort, the median OS was 36 (IQR: 22–62) months. The 1-year, 3-year, and 5-year DFS rates were 68.8%, 33.5%, and 25.6%, respectively. The 1-year, 3-year, and 5-year OS rates were 89.3%, 58.6%, and 44.1%, respectively. The Kaplan–Meier survival curve is shown in **Figures 1A, B**.

In the validation cohort, the median OS was 41 (IQR: 17–62) months. The 1-year, 3-year, and 5-year CSS rates were 86.7%, 64.7%, and 56.0%, respectively. The 1-year, 3-year, and 5-year OS rates were 85.3%, 58.6%, and 47.7%, respectively. The Kaplan–Meier survival curve is shown in **Figures 1C, D**.

### Survival Analysis for the Training Cohort

Univariate analysis revealed that age (≥69 years), tumor size (≥2.7 cm), poor differentiation, T3 stage, LNR (>0.1), and blood vessel invasion were risk factors for OS in AC patients, while tumor size (≥2.7 cm), poor differentiation, T3 stage, the number of total regional examined lymph nodes (<6), LNR (>0.1), and blood vessel invasion were risk factors for DFS in AC patients. Multivariate analysis showed that only LNR (>0.1) (HR: 2.557, 95% CI: 1.377–4.747, *p* = 0.003) was the independent risk factor for OS in AC patients, while T3 stage (HR: 3.654, 95% CI: 1.290–10.350, *p* = 0.015) and LNR (>0.1) (HR: 2.418, 95% CI: 1.352–4.324, *p* = 0.003) were independent risk factors for DFS in AC patients. The detailed results of Cox regression were demonstrated in **Tables 2** and **3**.

**Table 2:** Univariate and multivariate Cox regression analyses of OS of the AC patients in the training cohort.

Characteristic	Univariable analysis		Multivariable analysis	
	HR [95% CI]	*p*-Value	HR [95% CI]	*p*-Value
Sex				
Male	Reference			
Female	0.908 [0.607–1.358]	0.638		
Age				
≤47	Reference		Reference	
48–68	1.359 [0.789–2.343]	0.269	1.349 [0.758–2.400]	0.308
≥69	2.091 [1.003–4.361]	0.049	1.356 [0.581–3.163]	0.481
Preoperative biliary drainage				
No	Reference			
Yes	0.964 [0.583–1.594]	0.887		
Intraoperative transfusion				
No	Reference			
Yes	0.844 [0.567–1.258]	0.405		
Operation time				
≤6 h	Reference			
>6 h	1.236 [0.803–1.905]	0.336		
Tumor size				
≦2.7 cm	Reference		Reference	
>2.7 cm	1.724 [1.145–2.595]	0.009	1.200 [0.750–1.921]	0.447
Differentiation				
Poor	Reference			
Moderate	0.937 [0.605–1.449]	0.769	0.855 [0.529–1.382]	0.522
Well	0.470 [0.260–0.849]	0.012	0.677 [0.330–1.391]	0.289
T stage				
T1	Reference		Reference	
T2	1.490 [0.686–3.235]	0.314	1.299 [0.562–3.001]	0.540
T3	3.319 [1.578–6.981]	0.002	2.230 [0.914–5.439]	0.078
Regional nodes examined				
≤6	Reference		Reference	
>6	1.453 [0.892–2.369]	0.134	1.109 [0.640–1.922]	0.712
LNR				
0	Reference		Reference	
≤0.1	1.740 [0.984–3.075]	0.057	1.471 [0.776–2.787]	0.236
>0.1	2.596 [1.588–4.245]	<0.001	2.557 [1.377–4.747]	0.003
Blood vessel invasion				
No	Reference		Reference	
Yes	1.587 [1.018–2.473]	0.042	1.015 [0.603–1.707]	0.956
Postoperative complications				
No	Reference		Reference	
Yes	1.487 [0.992–2.227]	0.054	1.519 [0.998–2.311]	0.051
Adjuvant treatment				
No	Reference		Reference	
Yes	1.353 [0.864–2.119]	0.186	0.613 [0.345–1.089]	0.095
Unknown	4.717 [1.704–13.054]	0.003	2.969 [0.861–10.236]	0.085

OS, overall survival; AC, ampullary carcinoma; LNR, lymph node ratio.

**Table 3:** Univariate and multivariate Cox regression analyses of DFS of the AC patients in the training cohort.

Characteristic	Univariable analysis		Multivariable analysis	
	HR [95% CI]	*p*-Value	HR [95% CI]	*p*-Value
Sex				
Male	Reference			
Female	0.967 [0.647–1.446]	0.872		
Age				
≤47	Reference			
48–68	1.283 [0.765–2.153]	0.345		
≥69	1.228 [0.551–2.737]	0.616		
Preoperative biliary drainage				
No	Reference			
Yes	0.889 [0.538–1.467]	0.644		
Intraoperative transfusion				
No	Reference			
Yes	0.822 [0.552–1.223]	0.333		
Operation time				
≤6 h	Reference			
>6 h	0.952 [0.605–1.497]	0.831		
Tumor size				
≦2.7 cm	Reference		Reference	
>2.7 cm	1.704 [1.134–2.561]	0.010	1.388 [0.879–2.190]	0.159
Differentiation				
Poor	Reference		Reference	
Moderate	1.016 [0.659–1.566]	0.944	0.957 [0.602–1.521]	0.854
Well	0.466 [0.255–0.851]	0.013	0.913 [0.415–2.012]	0.822
T stage				
T1	Reference		Reference	
T2	1.874 [0.783–4.485]	0.158	2.461 [0.937–6.461]	0.067
T3	4.372 [1.881–10.161]	0.001	3.654 [1.290–10.350]	0.015
Regional nodes examined				
≤6	Reference		Reference	
>6	0.601 [0.374–0.968]	0.036	0.610 [0.356–1.046]	0.072
LNR				
0	Reference		Reference	
≤0.1	1.368 [0.803–2.330]	0.249	1.274 [0.727–2.231]	0.398
>0.1	2.436 [1.483–4.002]	<0.001	2.418 [1.352–4.324]	0.003
Blood vessel invasion				
No	Reference		Reference	
Yes	1.802 [1.168–2.779]	0.008	0.928 [0.557–1.546]	0.774
Postoperative complications				
No	Reference			
Yes	1.306 [0.866–1.971]	0.203		
Adjuvant treatment				
No	Reference		Reference	
Yes	1.436 [0.939–2.198]	0.095	0.662 [0.367–1.054]	0.078
Unknown	Not available	0.966	Not available	0.960

DFS, disease-free survival; AC, ampullary carcinoma; LNR, lymph node ratio.

**Figure 2:**
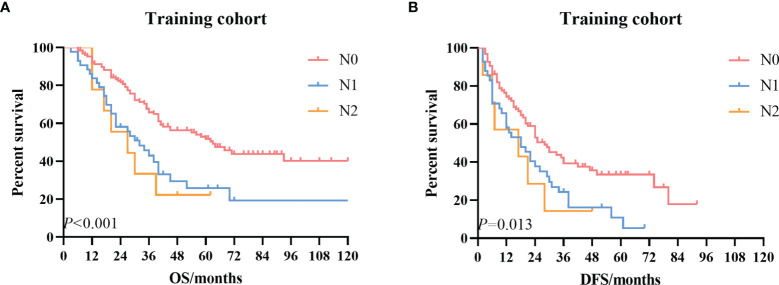
The Kaplan–Meier survival curves according to the N stage. **(A)** OS curve in the training cohort. **(B)** DFS curve in the training cohort. OS, overall survival; DFS, disease-free survival.

Then we conducted survival analysis including the variables age, tumor size, differentiation, T stage, N stage, blood vessel invasion, postoperative complications, and adjuvant treatment. The univariate survival curves based on the N stage are depicted in **Figure 2**. The multivariate analysis results demonstrated no significant difference in the survival (**Table 4**).

**Table 4:** Multivariate Cox regression analyses contained N stage in the AC patients.

Characteristic	OS		RFS	
	HR [95% CI]	*p*-Value	HR [95% CI]	*p*-Value
Age				
≤47	Reference			
48–68	1.288 [0.726–2.287]	0.387		
≥69	1.355 [0.580–3.164]	0.483		
Tumor size				
≦2.7 cm	Reference		Reference	
>2.7 cm	1.223 [0.757–1.975]	0.411	1.305 [0.823–2.072]	0.258
Differentiation				
Poor	Reference		Reference	
Moderate	0.861 [0.531–1.396]	0.544	0.970 [0.607–1.550]	0.900
Well	0.694 [0.337–1.427]	0.32	1.013 [0.467–2.200]	0.973
T stage				
T1	Reference		Reference	
T2	1.319 [0.571–3.045]	0.517	2.660 [1.011–6.995]	0.047
T3	2.212 [0.903–5.418]	0.082	3.997 [1.412–11.314]	0.009
Regional nodes examined				
≤6	Reference		Reference	
>6	1.032 [0.596–1.785]	0.911	0.527 [0.314–0.882]	0.015
N stage				
0	0.450 [0.177–1.141]	0.093	0.451 [0.173–1.177]	0.104
1	0.843 [0.342–2.081]	0.712	0.735 [0.287–1.884]	0.522
2	Reference		Reference	
Blood vessel invasion				
No	Reference		Reference	
Yes	1.082 [0.647–1.811]	0.763	1.019 [0.623–1.667]	0.939
Postoperative complications				
No	Reference			
Yes	1.539 [1.013–2.339]	0.044		
Adjuvant treatment				
No	Reference		Reference	
Yes	0.648 [0.364–1.156]	0.142	0.649 [0.381–1.104]	0.111
Unknown	3.032 [0.828–11.109]	0.094		

AC, ampullary carcinoma; OS, overall survival; RFS, recurrence-free survival.

### Proposed a New Lymph Node Ratio-Based Staging System

**Figure 3:**
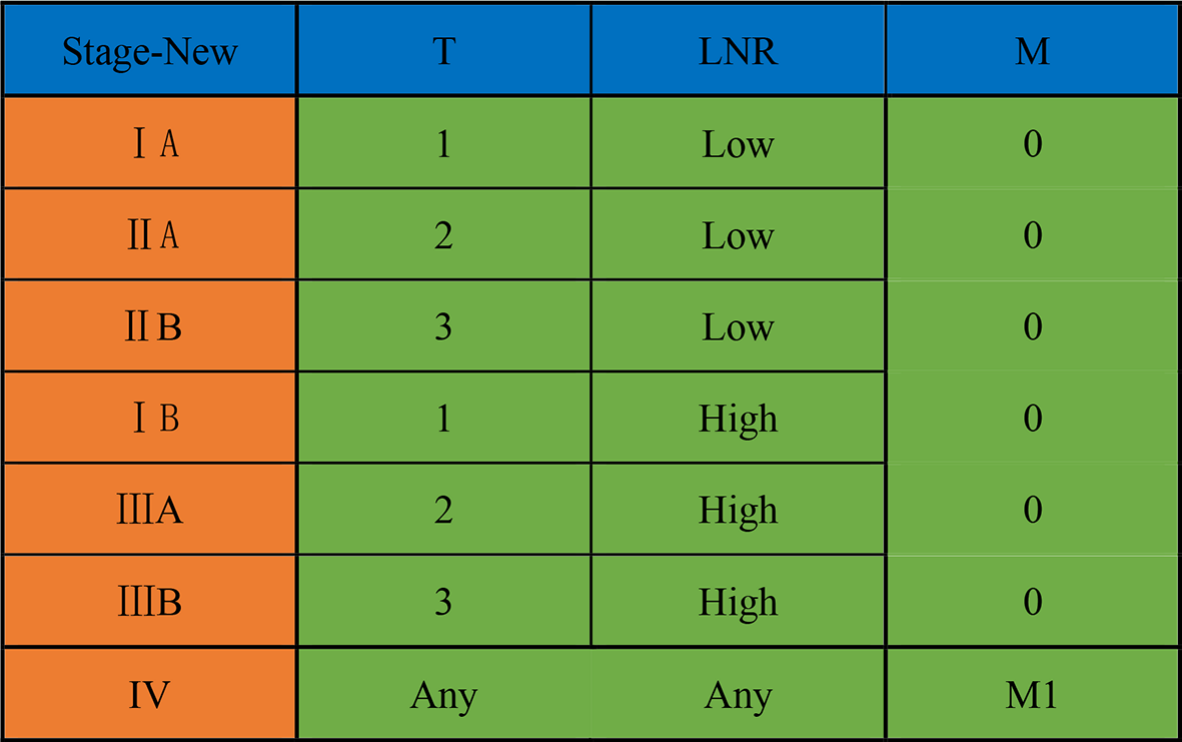
A new LNR-based staging system for ampullary carcinoma after curative pancreaticoduodenectomy. LNR, lymph node ratio.

Based on the results of survival analysis, we developed a new LNR-based staging system for AC after curative pancreaticoduodenectomy. Patients were divided into LNR-low stage and LNR-high stage in the new LNR-based staging system, considering that there was no significant survival difference between the LNR = 0 group and 0 < LNR ≤ 0.1 group. The detailed new staging system is illustrated in **Figure 3**.

### Validation of the New Lymph Node Ratio-Based Staging System

According to the new staging system, we then re-staged the patients in the training cohort and validation cohort. Under the new staging system, the number of patients in the training cohort and validation cohort of stage I, stage II, and stage III was 30 (11%) vs. 18 (9%), 190 (72%) vs. 96 (48%), and 44 (17%) vs. 85 (43%), respectively. The detailed proportions are demonstrated in **Supplementary Figure 3**.

**Figure 4:**
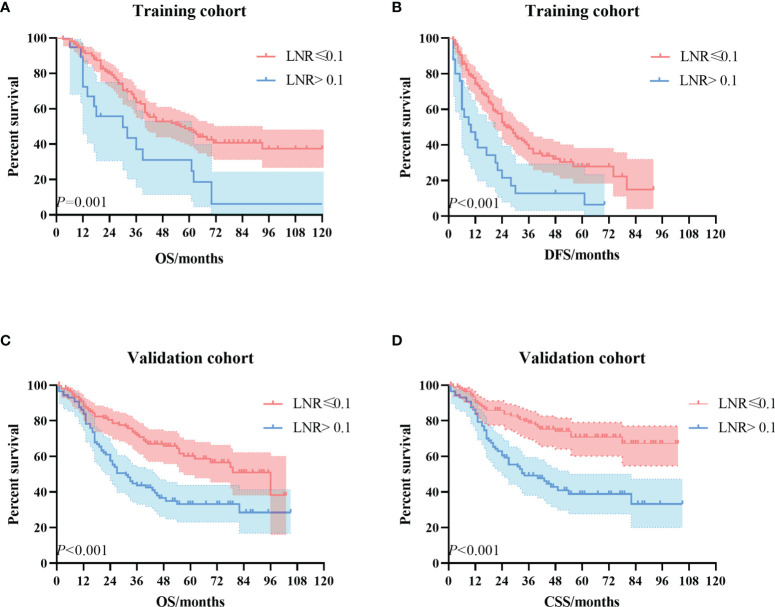
The Kaplan–Meier survival curves according to the LNR stage. **(A)** OS curve in the training cohort. **(B)** DFS curve in the training cohort. **(C)** OS curve in the validation cohort. **(D)** CSS curve in the validation cohort. LNR, lymph node ratio; OS, overall survival; DFS, disease-free survival; CSS, cancer-specific survival.

**Figure 5:**
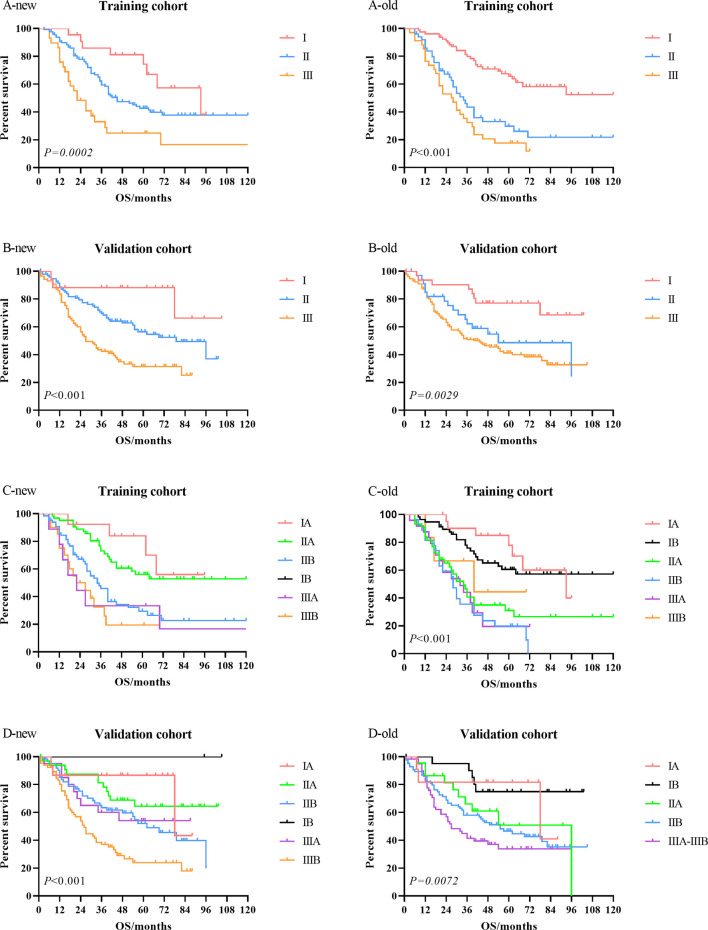
Comparison of the Kaplan–Meier survival curves between LNR-based staging system (new) and current 8th AJCC TNM staging system (old) in the training cohort and validation cohort. LNR, lymph node ratio; AJCC, American Joint Committee on Cancer.

Kaplan–Meier univariate survival curves stratified by LNR in the training cohort and validation cohort are depicted in **Figure 4** and demonstrated a significant difference in survival (*p* ≤ 0.001). Subsequently, we compared the new stage and the current 8th AJCC TNM stage in the training cohort and validation cohort. The results revealed that the survival curves of different tumor stages could clearly be distinguished from each other (**Figure 5**).

## Discussion

The current study demonstrated that LNR was a more powerful prognostic factor than the current N stage according to the 8th AJCC staging system. We established a new staging system by substituting LNR stage for the N stage and divided LNR into LNR-low stage and LNR-high stage with the best cutoff value of 0.1. By comparing the survival curves of the training cohort and the validation cohort, we found that the LNR-based staging system had better clinical benefits than the 8th AJCC TNM staging system.

LNR has been confirmed as a prognostic factor of AC patients, but no uniform cutoff threshold for LNR has been established. Kim et al. retrospectively analyzed 71 patients with AC who underwent adjuvant chemotherapy after radical resection and found that LNR > 0.15 was an independent risk factor for OS (10). In an analysis of 212 AC patients who received radical surgery in Taiwan, LNR > 0.056 indicated poor DFS and OS (11). Similarly, a study by Falconi et al. explored multiple cutoff values (LNR = 0, 0 < LNR ≤ 0.2, 0.2 < LNR ≤ 0.4, LNR > 0.4) in AC patients and found that high LNR was associated with OS (23). Moreover, a high LNR (≥0.15) was closely associated with decreased time to distant recurrence than low LNR (LNR = 0, and 0.01 ≤ LNR ≤ 0.14) in AC patients after surgery (18). However, some previous studies revealed that LNR was not an independent significant prognostic factor in AC patients (16, 19, 21). In the present study, we confirmed LNR as a significant prognostic factor in AC patients after curative pancreaticoduodenectomy and estimated the cutoff value of 0.1. Such a difference in the cutoff value of LNR might be related to several factors. Firstly, the difference in social characteristics and ethnicity across studies may responsible for this discrepancy. Secondly, dissection of the lymph nodes was the major factor influencing the total number of resected lymph nodes and LNR. In the present study, we only included AC patients after curative pancreaticoduodenectomy and excluded other radical surgery modes. Thirdly, the difference in the follow-up time and adjuvant treatment in each study may also affect the results.

In the current 8th AJCC staging system, the number of metastatic lymph nodes is the basis of N stage. The prognostic value of different numbers of LNM has been explored in many studies (1, 12, 13, 15). It was worth noting that in a recent study, the investigators demonstrated that nearly 16.8% AC patients with pathologic negative lymph nodes were estimated to have undetected LNM through nodal staging score (6). Meanwhile, patients with a high nodal staging score generally had longer OS (6). However, in our cohort, we found the current N stage was not an independent prognostic factor for AC patients. A possible explanation is that the extent of lymphadenectomy and the primary sites of metastatic lymph nodes were also crucial factors for survival (1).

Currently, no consensus has been reached regarding the lymphadenectomy for the AC patients due to limited cases. The AJCC recommends that at least 12 lymph nodes should be dissected for AC patients (1). Some previous studies have revealed that an increasing number of resected lymph nodes might improve the positive rate of lymph nodes and prolong the survival time (8, 12, 17). In the present study, we found no significant relationship between the total number of lymph nodes and prognosis. Several reasons might account for this apparent discrepancy. Firstly, the location of metastatic lymph nodes of AC patients may be different in different studies. A recent study demonstrated that the prognosis was worse in AC patients with regional lymph nodes metastasis in other sites than only in the pancreatic head region (1). Secondly, in our study, more AC patients have negative lymph nodes and early pathologic stage. Therefore, the survival may not reach statistical differences.

On the basis of our analysis, we established a new staging system-based LNR and replaced the current 8th AJCC N staging for the first time. The new LNR-based staging system shows certain advantages over the 8th AJCC TNM staging system in both the training cohort and validation cohort. Under the new staging system, LNR is used to correlate LNM and surgical dissection quality. On the one hand, even if the numbers of metastatic lymph nodes are high in some AC patients, they are in the LNR-low staging due to thorough lymph node dissection. On the other hand, the new system avoids to a certain extent the incomplete lymph node dissection leading to a lower N stage of AC patients who have the LNR-high stage in fact. However, the new LNR-based staging system still needs to be validated in future larger prospective cohorts.

To our knowledge, this single-center cohort study has the largest size evaluating LNR in AC patients after curative pancreaticoduodenectomy. Moreover, we incorporated LNR into the staging system for the first time and established a new staging system. In order to verify the clinical benefit of the new LNR-based staging system, we used data from the SEER database for validation. Despite these strengths, we acknowledged several potential limitations that should be considered objectively. Firstly, this was a single-center retrospective study with a limited number of patients and clinical variables. Secondly, some missing important clinical data, such as adjuvant treatment and the levels of tumor biomarkers, might have a certain impact on the results of this study. Thirdly, some patients had shorter follow-up time, and the rate of loss to follow-up was relatively high.

## Conclusions

In conclusion, the present study demonstrated that LNR was an independent prognostic factor in AC patients after curative pancreaticoduodenectomy. Survival analysis revealed that the new LNR-based staging system had better clinical benefits than the 8th AJCC TNM staging system. However, further prospective studies with larger patients are necessary to validate the new LNR-based staging system.

## Data Availability Statement

The raw data supporting the conclusions of this article will be made available by the authors, without undue reservation.

## Ethics Statement

Ethical review and approval were not required for the study on human participants in accordance with the local legislation and institutional requirements. Written informed consent for participation was not required for this study in accordance with the national legislation and the institutional requirements.

## Author Contributions

1) Guarantor of integrity of the entire study: XC and DZ. 2) Study concepts and design: XZ and DZ. 3) Provision of study materials or patients: XZ, CS, and ZL. 4) Collection and assembly of data: XZ, LZ, PN, TW, XC, YC, and CG. 5) Statistical analysis: XZ. 6) Manuscript preparation: all authors. 7) Manuscript editing: all authors.

## Conflict of Interest

The authors declare that the research was conducted in the absence of any commercial or financial relationships that could be construed as a potential conflict of interest.

## Publisher’s Note

All claims expressed in this article are solely those of the authors and do not necessarily represent those of their affiliated organizations, or those of the publisher, the editors and the reviewers. Any product that may be evaluated in this article, or claim that may be made by its manufacturer, is not guaranteed or endorsed by the publisher.

## Supplementary Material

Supplementary Figure 1 The optimal cut-off value of LNR according to the X-tile software.

Supplementary Figure 2 The optimal cut-off value of total number of lymph nodes examined according to the X-tile software.

Supplementary Figure 3 The proportions of patients in the training cohort and validation cohort under the new staging system.
