# Supplementary figures and images for: Development and Validation of a New Lymph Node Ratio-Based Staging System for Ampullary Carcinoma After Curative Pancreaticoduodenectomy
Source: Front Oncol. 2022 Jan 20;11:811595. doi: 10.3389/fonc.2021.811595 (PMC8810493; doi:10.3389/fonc.2021.811595)

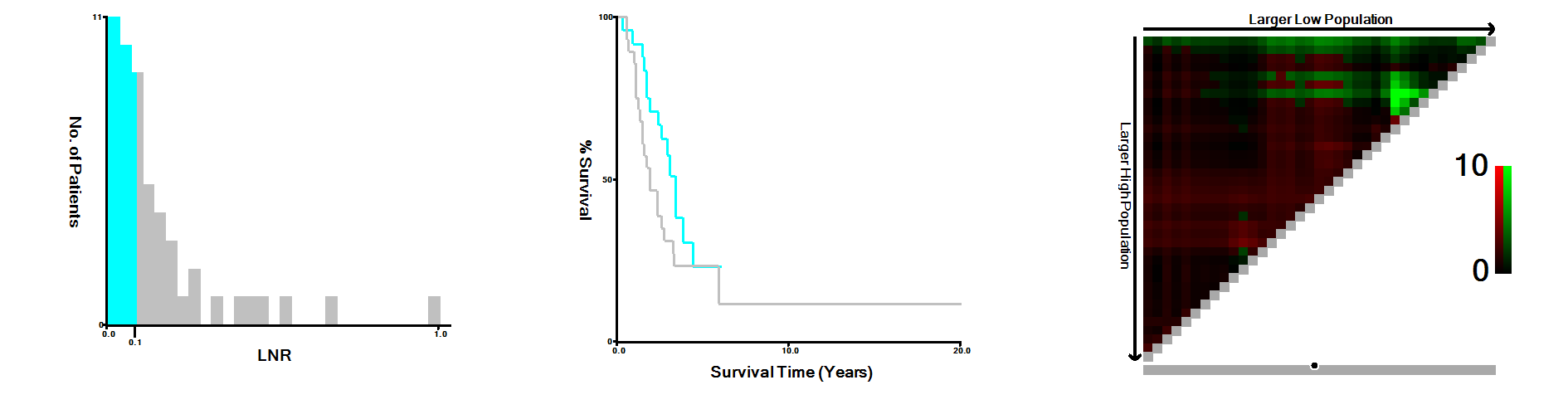

Supplement: Supplementary Figure 1 — The optimal cut-off value of LNR according to the X-tile software. [file Image_1.tif]

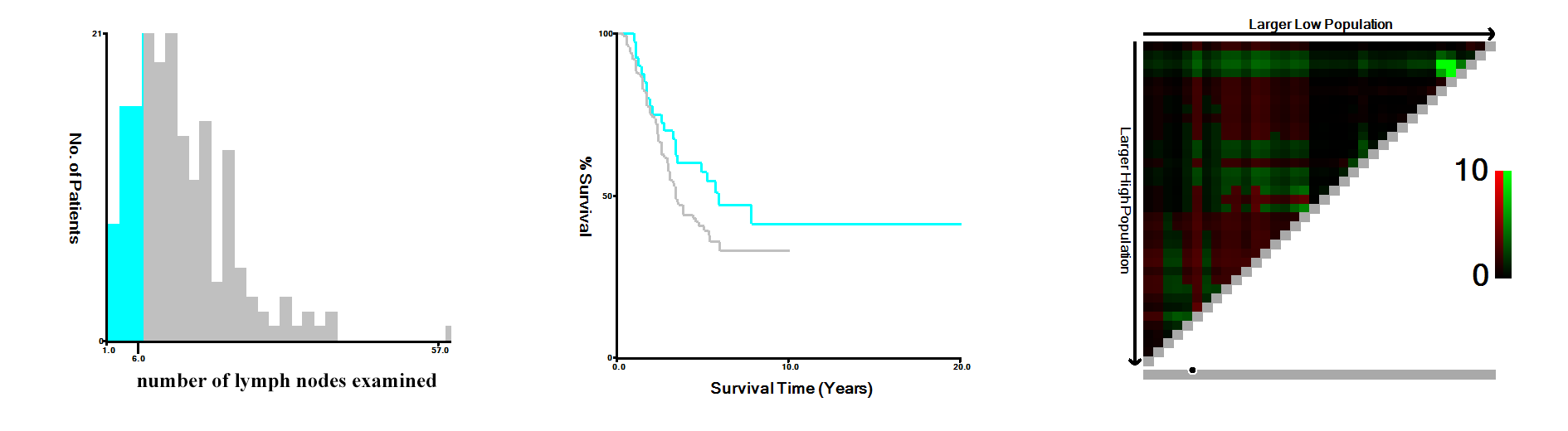

Supplement: Supplementary Figure 2 — The optimal cut-off value of total number of lymph nodes examined according to the X-tile software. [file Image_2.tif]

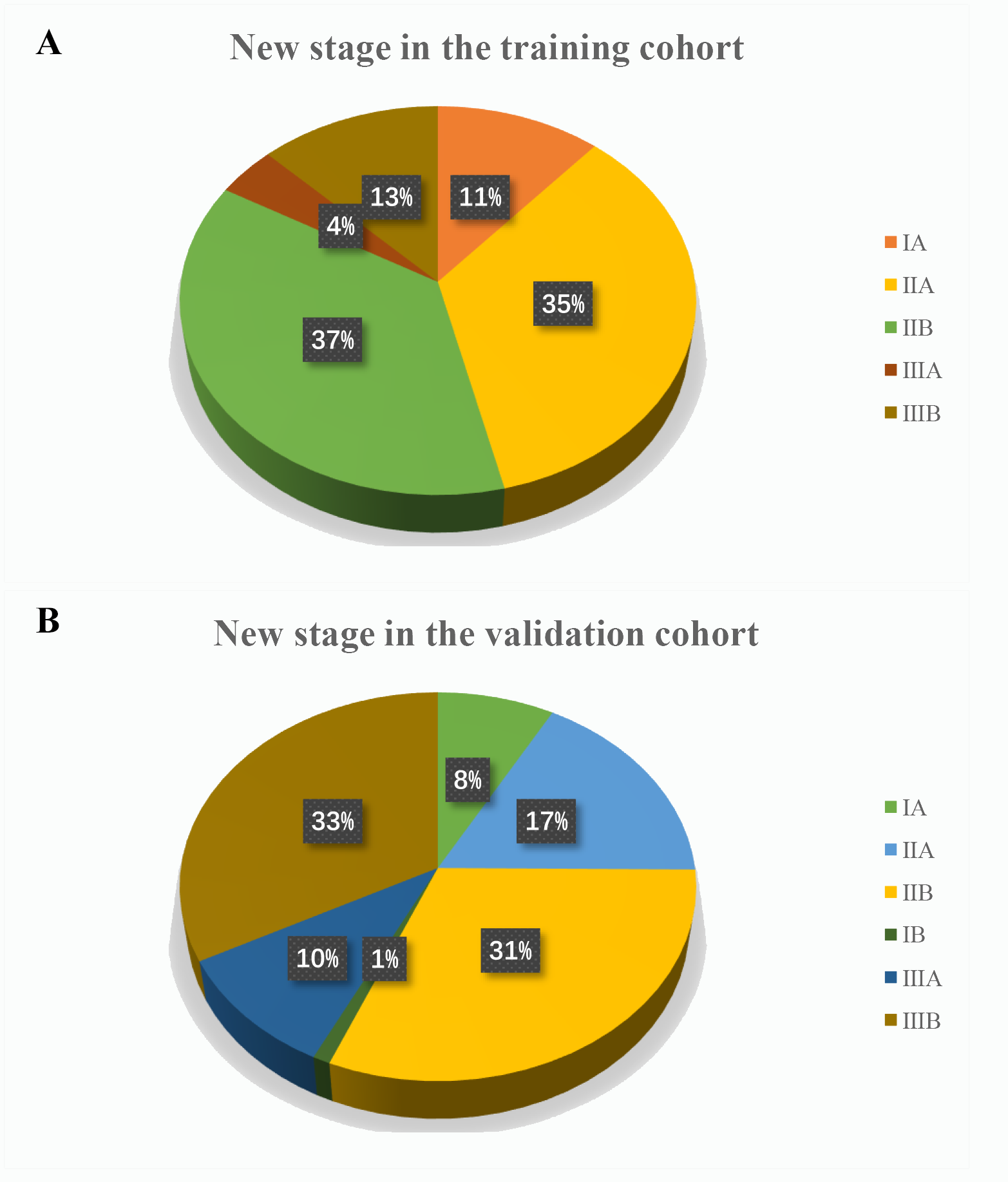

Supplement: Supplementary Figure 3 — The proportions of patients in the training cohort and validation cohort under the new staging system. [file Image_3.tif]
